# Supplementary material for: Anomalous magnetoresistance due to longitudinal spin fluctuations in a Jeff = 1/2 Mott semiconductor
Source: Nat Commun. 2019 Nov 22;10:5301. doi: 10.1038/s41467-019-13271-6 (PMC6874576; doi:10.1038/s41467-019-13271-6)
Supplement: Supplementary file 1 — Supplementary Information [file 41467_2019_13271_MOESM1_ESM.pdf]

Supplementary Information

Anomalous Magnetoresistance due to Longitudinal Spin Fluctuations in a J_{eff} = 1/2 Mott Semiconductor

Hao et.al

Supplementary Figures

1) Demonstration of the J_{eff} = 1/2 state

As shown in Supplementary Figure 1(a), the XAS spectra reveals a strong absorption peak at Energy = 11.22 keV, confirming the Ir^{4+} valence state[1]. Around the Ir L_3-edge, an appreciable XMCD effect was identified with the value maximizing at energy ~3 eV lower than the absorption edge, which reveals the dominant role of the Ir moments in the net magnetization. Showing in Supplementary Figure 1(b) is the XMCD measurement around the Ir L_2-edge, where no observable signal has been found. Since the electron excitation between the $2p_{1/2}$ and J_{eff} = 1/2 state is symmetrically forbidden[2], the absence of XMCD effect around the Ir L_2-edge confirms that we have realized the J_{eff} = 1/2 state in the SL, indicating that the low-energy physics can be captured with the pseudo-spin-half Hubbard model[3,4].

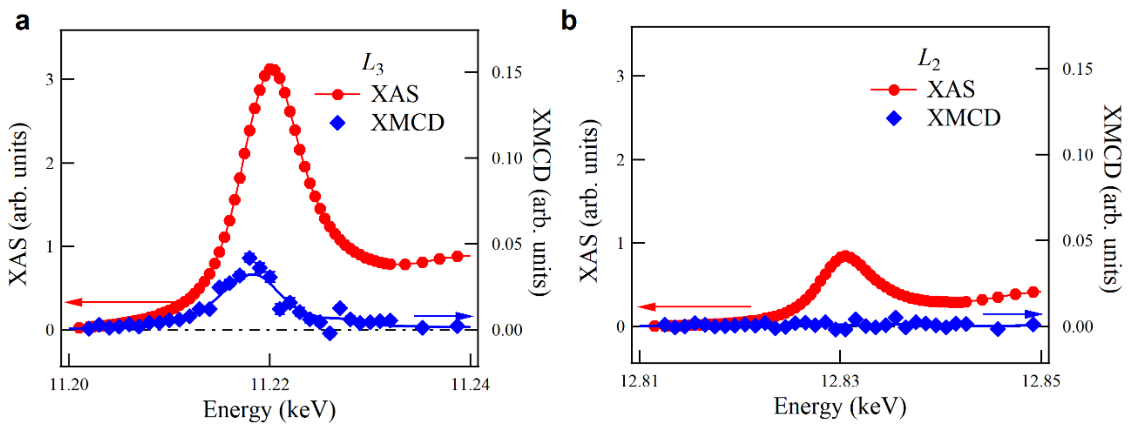

Supplementary Figure 1. X-ray absorption spectroscopy (XAS) and XMCD measurements of the SL. Energy dependent XAS and XMCD around the Ir L_3-edge (a) and Ir L_2-edge (b) at 10 K. The XMCD signal is the average of two independent measurements in opposite magnetic field of 1 T in order to eliminate magnetic field related artifacts.

2) Magnetization measurements

We measured macroscopic magnetic properties of the SL along the in-plane as well as the out-of-plane direction. As shown in Supplementary Figure 2, a sharp upturn of magnetization can be clearly seen in both directions for the SL when cooling below 150 K, in consistent with the AFM transition characterized through magnetic resonant scattering experiment and XMCD measurement (see Fig. 1f in the main text). The anisotropic ratio of the in-plane and out-of-plane components can be estimated to be about 10 at 50 K.

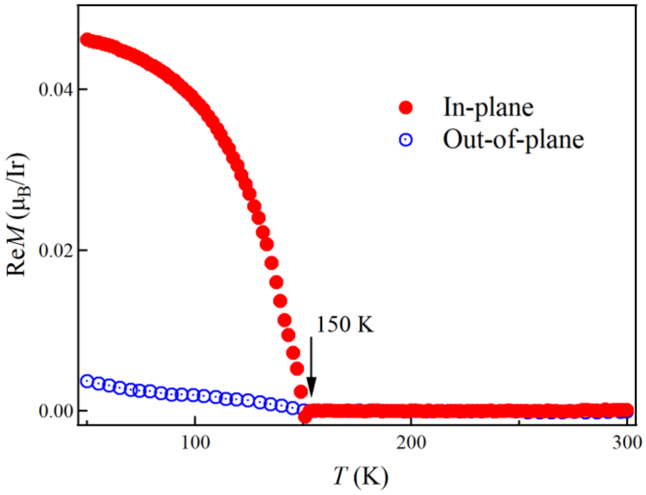

Supplementary Figure 2. Anisotropic magnetization measurements. Temperature dependent in-plane (Full) and out-of-plane (open) remnant magnetizations. The remnant magnetization was measured during zero-field warming process after cooling the sample in a 0.5 T in-plane magnetic field.

3) Uniform susceptibility extracted from XMCD measurement

We utilized XMCD (Ir L_3-edge) to capture the in-plane uniform magnetization. Following the XMCD spectra shown in Supplementary Figure 1(a), the x-ray energy was fixed at the value that maximizes the XMCD intensity. Then the thermal evolution of the XMCD intensity was

recorded under different magnetic fields. As shown in Supplementary Figure 3(a), the zero-field XMCD well follows the temperature dependence of the zero-field magnetization, demonstrating the net magnetization origin of the XMCD intensity. Under a 5T in-plane magnetic field, the XMCD shows a clear enhancement as compared to the zero-field counterpart when temperature cooling toward the transition temperature, after which the two sets of XMCD data get closer to each other. Showing in Supplementary Figure 3(b) are two XMCD spectra under 0 T and 5 T at 100 K. There is no clear field-induced change in the integrated intensity at 100 K within the experimental error, confirming the observation in Supplementary Figure 3(a). Thus, the temperature dependent uniform susceptibility can be deduced from the in-plane field-induced difference of the XMCD between 5 and 0 T after properly scaling with the magnetization data.

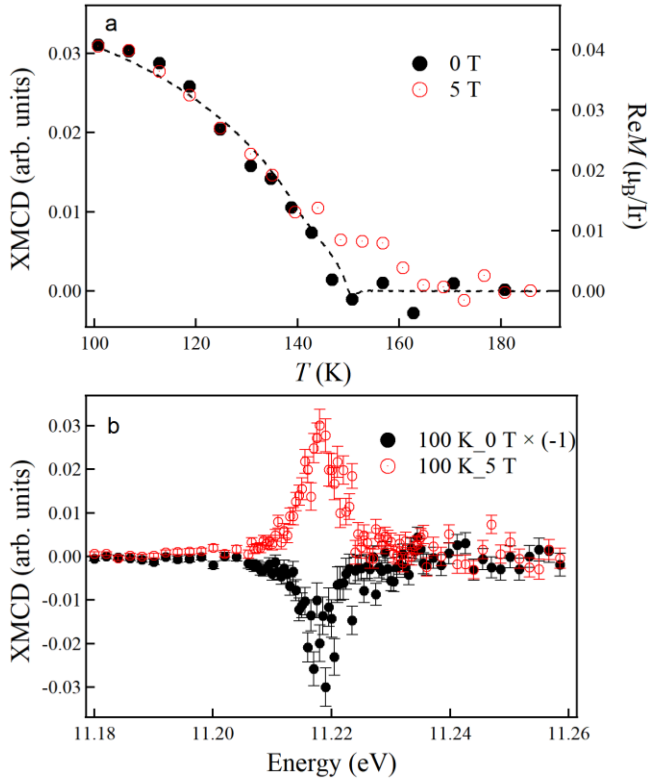

Supplementary Figure 3. Temperature dependent XMCD under different magnetic fields. (a) XMCD intensity (Ir L_3-edge) as a function of temperature under in-plane magnetic fields of 0 T (black circle) and 5 T (red circle). Before measuring the zero-field XMCD during warming process, to properly compare with the zero-field magnetization (dashed curve), the sample was cooled down under the same magnetic field as that used in the remnant magnetization measurement, i.e., a 0.5 T in-plane magnetic field. (b) XMCD spectra at 100 K under in-plane magnetic fields of 0 T (black circle) and 5 T (red circle). Magnetic field related artifacts were eliminated by averaging data that measured in opposite magnetic fields.

4) Magnetic domain related effects in MR measurements

To capture magnetic domain related effects in our MR measurements, we first change magnetic domain population by cooling the sample in different magnetic fields. Then, the sample resistance was recorded as a function of in-plane magnetic field at different temperatures. As shown in Supplementary Figure 4, at temperatures below T_N (red), a clear hysteresis-like behavior can be seen in the field dependence of resistance measured after cooling the sample in zero magnetic field (ZFC), indicating an initial multidomain structure. On the other hand, the low-field hysteresis becomes non-observable when cooling the sample under an 8T in-plane magnetic field (FC), suggesting of a single-domain state. The difference of the ZFC- and FC-curves is thus characteristic of domain wall contributions to the total MR. The magnetic domain walls contribute about 0.2% in the total MR at 110 K, and the contribution drops to 0 at 150 K. The diminished magnetic domain wall related effect at 150 K is understandable since there are no well-defined magnetic domains at temperatures above T_N.

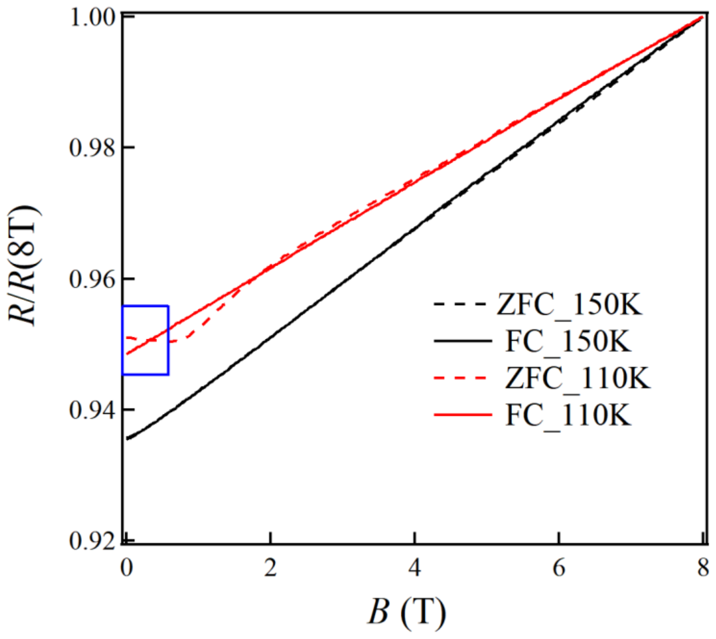

Supplementary Figure 4. In-plane magnetic field dependent resistance at different temperatures. The resistance was measured with the same setup after cooling the sample under zero magnetic field (ZFC) or an 8 T in-plane magnetic field (FC). The low-field resistance difference is highlighted in a blue rectangle.

5) Anisotropic magnetoresistance

To study in-plane anisotropy of the anomalous MR, we measured MR with magnetic field along the STO [100] and [110] directions. As shown in Supplementary Figure 5, an anisotropic effect can be clearly seen. Nonetheless, MR in both directions display the similar temperature dependence and does not change the conclusion of the main result.

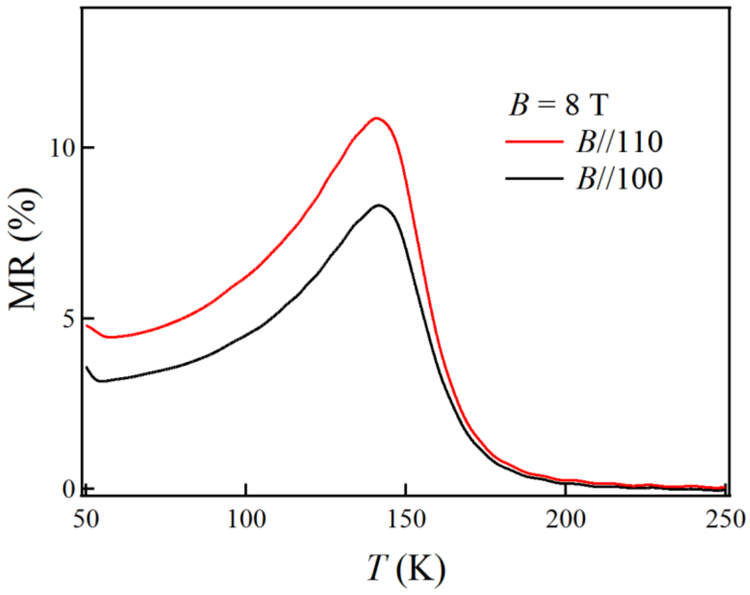

Supplementary Figure 5. Anisotropic MR measurements. Temperature dependent MR with an in-plane magnetic field applied along the STO [110] (red) or [100] (black) direction.

6) Anomalous magnetoresistance in $[(SrIrO_3)_2/(SrTiO_3)_1]$ superlattice

We have grown another heterostructure, $[(SrIrO_3)_2/(SrTiO_3)_1]$, on $SrTiO_3$ substrate[5]. The obtained superlattice hosts a canted *c*-axis AFM structure and displays a resistance anomaly around the magnetic transition temperature. Supplementary Figure 6 shows temperature dependent in-plane MR and remnant magnetization. An anomalous MR behavior has been observed around the magnetic transition, similar to the observation in the sister superlattice $[(SrIrO_3)_1/(SrTiO_3)_1]$ in the main text.

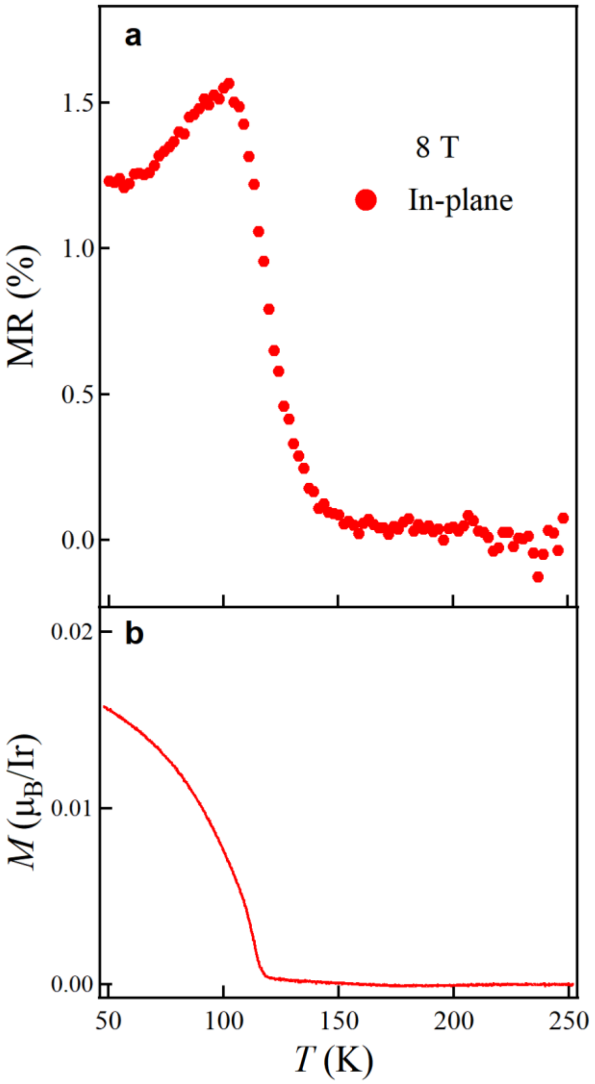

Supplementary Figure 6. (a) Temperature dependent MR under an 8 T in-plane magnetic field of $[(SrIrO_3)_2/(SrTiO_3)_1]$ grown on a $SrTiO_3$ (001) substrate. (b) Temperature dependence of the in-plane magnetization.

7) Resonant enhancement of magnetic scattering intensity

Supplementary Figure 7 shows the energy profile around the Ir L_3-edge for the (0.5 0.5 2) magnetic reflection. A strong intensity enhancement can be clearly seen on the SL, with the maximum at energy about 3 eV lower than the Ir L_3 absorption edge (Energy = 11.22 keV), which

characterizes the resonant effect of Ir moment[1,6,7]. The temperature scan and *L*-scan measurements as shown in the main text were performed with the x-ray energy siting on the resonant peak.

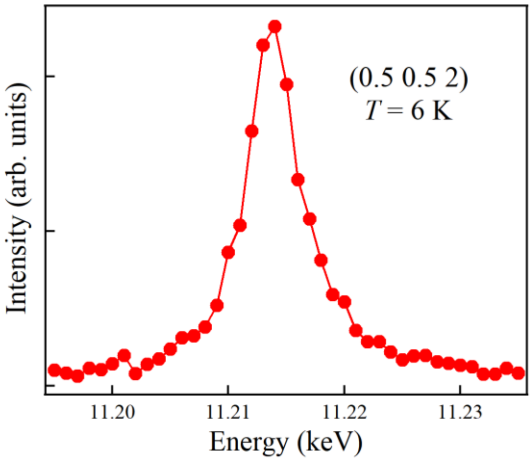

Supplementary Figure 7 Magnetic resonant scattering measurements. Energy scan of the magnetic reflection (0.5 0.5 2) of the SL around the Ir L_3-edge at 6 K.

Supplementary References

1 Kim, B. J. *et al.* Phase-Sensitive Observation of a Spin-Orbital Mott State in Sr_2IrO_4. *Science* **323**, 1329-1332 (2009).

2 Kim, B. J. *et al.* Novel J_{eff}=1/2 Mott state induced by relativistic spin-orbit coupling in Sr_2IrO_4. *Phys. Rev. Lett.* **101**, 076402 (2008).

3 Jackeli, G. & Khaliullin, G. Mott Insulators in the Strong Spin-Orbit Coupling Limit: From Heisenberg to a Quantum Compass and Kitaev Models. *Phys. Rev. Lett.* **102**, 017205 (2009).

4 Wang, F. & Senthil, T. Twisted Hubbard Model for Sr_2IrO_4: Magnetism and Possible High Temperature Superconductivity. *Phys. Rev. Lett.* **106**, 136402 (2011).

5 Hao, L. *et al.* Two-Dimensional J_{eff}=1/2 Antiferromagnetic Insulator Unraveled from Interlayer Exchange Coupling in Artificial Perovskite Iridate Superlattices. *Phys. Rev. Lett.* **119**, 027204 (2017).

6 Kim, J. W. *et al.* Dimensionality Driven Spin-Flop Transition in Layered Iridates. *Phys. Rev. Lett.* **109**, 037204 (2012).

7 Boseggia, S. *et al.* Robustness of Basal-Plane Antiferromagnetic Order and the J_{eff} =1/2 State in Single-Layer Iridate Spin-Orbit Mott Insulators. *Phys. Rev. Lett.* **110**, 117207 (2013).
